# Serum albumin, NLR and PLR in pre-treatment assessment of bronchiectasis exacerbations

**DOI:** 10.6026/973206300223236

**Published:** 2026-06-30

**Authors:** Reetu G, Vinod Kumar Viswanathan

**Affiliations:** 1 Department of Respiratory Medicine, Institute of Thoracic Medicine, Madras Medical College, the Tamilnadu Dr. MGR Medical University, Chennai, Tamilnadu, India

**Keywords:** Albumin, bronchiectasis, neutrophil lymphocyte ratio (NLR), platelet lymphocyte ratio (PLR), severity

## Abstract

Bronchiectasis severity scores, through useful in disease progression, are inadequate for monitoring exacerbation treatment. Therefore, it is of interest to investigate the correlation between serum albumin, Platelet Lymphocyte Ratio (PLR) and Neutrophil Lymphocyte Ratio (NLR) with disease severity in exacerbated bronchiectasis patients. Serum albumin and NLR demonstrated significant correlation with disease severity, while PLR did not. Post-treatment, both markers showed improvement, indicating their role in treatment monitoring. Thus, we show NLR and serum albumin as reliable biomarkers for assessing severity and monitoring treatment response in bronchiectasis.

## Background:

Bronchiectasis is a suppurative lung disease with heterogeneous phenotypic features. It is recognized as a chronic respiratory condition characterized by abnormal and permanent dilation of the bronchi, which results from inflammatory injury to the airways [1]. This damage causes a loss of structural integrity in the muscle, elastic tissue and cartilage of the bronchi. Clinically, bronchiectasis is associated with chronic symptoms, including persistent cough, sputum production, exertional dyspnea and recurrent lower respiratory tract infections [2]. These symptoms represent the final common pathway for various disease processes, leading to bronchial dilatation and chronic inflammation. The disease manifests through different clinical patterns and it can be caused by a variety of underlying conditions, such as infections, autoimmune diseases, or genetic disorders [3]. The structural damage caused by chronic inflammation disrupts the normal functioning of the airways, making them prone to infection and mucus accumulation. The continuous cycle of infection and inflammation results in the progression of the disease, leading to severe impairment of lung function [4]. To assess the severity of bronchiectasis, several scoring systems have been developed, including the FACED and Bronchiectasis Severity Index (BSI) scores. These scoring systems take into account a variety of clinical and radiological factors, such as forced expiratory volume in one second (FEV1), age, sputum culture and the presence of comorbidities [5]. However, the FACED score has limitations, particularly in that it does not account for exacerbations, which are considered by many clinicians to be one of the most important modifiable markers of severity [6]. In fact, prior exacerbations are the strongest predictor of future exacerbations, highlighting the importance of identifying and managing exacerbations early on. This limitation has led to the preference for the BSI score in clinical practice, as it better incorporates exacerbations into the assessment of disease severity [7]. Despite the use of these scoring systems, there remains a significant challenge in monitoring disease progression and treatment efficacy. Current methods for evaluating the disease primarily rely on clinical symptoms, radiographic findings and exacerbation frequency, but these tools are not always sufficient in tracking the nuances of disease progression, particularly in terms of treatment response [8]. Biomarkers, which are measurable indicators of the presence or severity of disease, have the potential to provide additional insights into the pathophysiology of bronchiectasis and help guide treatment decisions [9]. Therefore, it is of interest to report the potential of biomarkers like NLR and serum albumin as practical, reliable and accessible methods for monitoring bronchiectasis, particularly during exacerbations and in evaluating the response to treatment.

## Methodology:

This prospective observational study included 110 patients admitted with bronchiectasis exacerbation. An exacerbation of bronchiectasis, in the absence of a specific diagnostic test, is defined as a deterioration in three or more of the following symptoms lasting at least 48 hours, accompanied by a change in treatment and the exclusion of other potential causes of clinical deterioration: Cough, sputum volume and/or consistency, sputum purulence, breathlessness and/or exercise intolerance, fatigue and/or malaise and hemoptysis. Patients presenting with exacerbations of bronchiectasis were diagnosed using high-resolution chest CT (HRCT) following the diagnostic criteria of the British Thoracic Society (BTS) guidelines. Bronchiectasis is defined by bronchial dilatation as suggested by one or more of the following criteria: broncho- arterial ratio >1 (internal airway lumen versus adjacent pulmonary artery), lack of tapering and airway visibility within 1 cm of the costal pleural surface or touching the mediastinal pleura. Commonly associated indirect signs include bronchial wall thickening, mucus impaction and mosaic perfusion/air trapping on expiratory CT. The following patients were excluded from the study: Those not willing to participate, patients with chronic kidney disease with a glomerular filtration rate (GFR) less than 60 ml/min/1.7/m^2^, those with hematological diseases, malignancy, immunocompromised patients and patients undergoing chemotherapy or cytotoxic drugs. Venous blood samples were obtained from patients upon admission, prior to starting antibiotic treatment and followed up after 2 weeks and 1 month of treatment. Hemogram measurements, including total white blood cells, neutrophils, lymphocytes and platelets, were measured. Biochemical analysis to measure serum albumin was carried out. The Platelet Lymphocyte Ratio (PLR) was calculated as the ratio of platelet count to lymphocyte count and the Neutrophil Lymphocyte Ratio (NLR) was calculated as the ratio of neutrophil count to lymphocyte count. The serum albumin, PLR and NLR were first measured at the time of admission and compared with the Bronchiectasis Severity Index (BSI) score to evaluate their correlation with disease severity. The above parameters were then measured post-treatment after 2 weeks and 1 month to evaluate their effectiveness as monitoring parameters for bronchiectasis. The collected data were analyzed using IBM SPSS Statistics for Windows, Version 23.0 (Armonk, NY: IBM Corp). Descriptive statistics, including frequency analysis and percentage analysis, were used for categorical variables, while mean and standard deviation (SD) were used for continuous variables. To identify significant differences in multivariate analysis, one-way ANOVA with Tukey's Post-Hoc test was used. Pearson Correlation was applied to assess the relationship between the variables. The Chi-Square test was used to find significance in categorical data. Statistical significance was set at p<0.05.

## Results:

The study included 110 patients, with a mean age of 53 ± 8 years. Males constituted 67.3% of the study population. These demographic characteristics are summarized in Table 1. Pre-treatment Neutrophil Lymphocyte Ratio (NLR) showed a statistically significant association with bronchiectasis severity as assessed by the Bronchiectasis Severity Index (BSI). The mean NLR increased progressively from the mild group to the severe group, with values of 1.41 ± 0.45, 1.73 ± 0.79, and 4.60 ± 2.42, respectively. This association was statistically significant with a p-value <0.005, as shown in Table 2. Pre-treatment serum albumin levels also demonstrated a significant relationship with disease severity. The mild group had the highest mean serum albumin level (3.85 ± 0.43 g/dL), followed by the moderate group (3.59 ± 0.43 g/dL), while the severe group had the lowest mean value (2.99 ± 0.51 g/dL). This inverse relationship between serum albumin and bronchiectasis severity was statistically significant with a p-value <0.005, as presented in Table 3. In contrast, the Platelet Lymphocyte Ratio (PLR) did not show a statistically significant association with BSI severity. The mean PLR values were 150.78 ± 66.17 in the mild group, 140.01 ± 55.24 in the moderate group, and 163.75 ± 72.46 in the severe group. The difference among the groups was not statistically significant, with a p-value of 0.419, as shown in Table 4. The trend of NLR following treatment showed improvement across all severity groups. In the mild group, mean NLR decreased from 1.41 ± 0.45 pre-treatment to 1.27 ± 0.31 at 2 weeks and 1.29 ± 0.20 at 1 month. In the moderate group, NLR decreased from 1.73 ± 0.79 to 1.52 ± 0.58 at 2 weeks and 1.44 ± 0.44 at 1 month. The severe group showed the most marked reduction, from 4.60 ± 2.42 pre-treatment to 2.36 ± 1.01 at 2 weeks and 1.59 ± 0.57 at 1 month. These findings indicate that NLR decreased following treatment, particularly in patients with severe bronchiectasis, as presented in Table 5. Serum albumin levels showed a progressive increase after treatment across all severity groups. In the mild group, serum albumin increased from 3.85 ± 0.43 g/dL pre-treatment to 3.99 ± 0.36 g/dL at 2 weeks and 4.10 ± 0.33 g/dL at 1 month. In the moderate group, levels increased from 3.59 ± 0.43 g/dL to 3.74 ± 0.37 g/dL at 2 weeks and 3.87 ± 0.31 g/dL at 1 month. In the severe group, serum albumin improved from 2.99 ± 0.51 g/dL pre-treatment to 3.25 ± 0.43 g/dL at 2 weeks and 3.49 ± 0.38 g/dL at 1 month. This improvement is shown in Table 6. Repeated measures ANOVA demonstrated statistically significant improvement in both NLR and serum albumin across all severity groups following treatment, with p <0.01. Overall, NLR and serum albumin were significantly associated with bronchiectasis severity and showed improvement after treatment, whereas PLR did not demonstrate a significant correlation with disease severity.

## Discussion:

Bronchiectasis is a chronic inflammatory disorder predominantly driven by neutrophilic airway inflammation, which is often exacerbated by increased numbers of neutrophils in the airways, as observed during exacerbations [10]. In our study, a significant association was found between the Neutrophil Lymphocyte Ratio (NLR) and disease severity based on the BSI score, with higher NLR values observed in severe cases. This finding is consistent with previous research where elevated NLR was shown to reflect a systemic inflammatory burden, particularly during exacerbations of bronchiectasis. Platelets interact with leukocytes and endothelial cells, releasing inflammatory factors that lead to the adhesion and migration of monocytes. This interaction links inflammation, thrombosis and atherogenesis, making Platelet Lymphocyte Ratio (PLR) an interesting marker [11]. However, in our study, PLR did not show a significant correlation with disease severity. Despite the theoretical relevance of PLR in inflammation, no significant differences in PLR were found across the severity groups, suggesting that PLR may not be a reliable biomarker for bronchiectasis severity. Georgakopoulou *et al.*(2020) [12] explored the value of NLR as a biomarker in bronchiectasis exacerbations and found that NLR values were significantly higher in patients with bronchiectasis exacerbations compared to healthy controls, which supports our findings. Similarly, in previous studies reference values for NLR (1.76) and PLR (120) were established, contributing to a deeper understanding of these biomarkers in both patients with bronchiectasis and the general population [13]. The link between chronic systemic inflammation and malnutrition, leading to hypoalbuminemia, is well-established. Our study demonstrated that serum albumin levels significantly correlated with the severity of bronchiectasis, with lower levels seen in more severe cases. These findings align with the literature, where hypoalbuminemia is often associated with disease severity and poor clinical outcomes. Low serum albumin levels, in particular, have been shown to reflect both systemic inflammation and poor nutritional status, indicating its potential as a useful monitoring parameter in bronchiectasis [14]. Several other studies have reinforced the role of serum albumin as a biomarker in bronchiectasis. Paliogiannis *et al.* (2021) [15] found that serum albumin levels strongly correlated with BSI scores, identifying it as an independent variable associated with disease severity. In another study by Ju *et al.* (2021) [16], low serum albumin levels were associated with higher severity scores and more frequent hospitalizations in bronchiectasis patients. In conclusion, our study supports the value of NLR and serum albumin as biomarkers for assessing disease severity and monitoring treatment responses in bronchiectasis. These biomarkers provide valuable insights into the pathophysiology of the disease and could serve as practical tools in clinical settings, particularly in resource-limited environments.

## Conclusion:

Neutrophil Lymphocyte Ratio (NLR) was significantly higher in patients with severe bronchiectasis and showed improvement post-treatment, while Platelet Lymphocyte Ratio (PLR) did not correlate with disease severity. Serum albumin levels were low in patients with severe disease and improved during follow-up, highlighting its role as a useful monitoring parameter. Both NLR and serum albumin are reliable biomarkers for assessing severity and treatment response in bronchiectasis, especially in resource-limited settings.

## Figures and Tables

**Table 1:** Demographic characteristics

**Parameter**	**Value**
Sample Size	110
Mean Age	53± 8 years
Male (%)	67.30%

**Table 2:** Pre-treatment NLR according to BSI severity

**Severity**	**Mean NLR± SD**	**p-value**
Mild	1.41± 0.45	
Moderate	1.73± 0.79	
Severe	4.60± 2.42	<0.005

**Table 3:** Pre-treatment serum albumin according to BSI severity

**Severity**	**Mean Albumin (g/dL)± SD**	**p-value**
Mild	3.85± 0.43	
Moderate	3.59± 0.43	
Severe	2.99± 0.51	<0.005

**Table 4:** Pre-treatment PLR according to BSI severity

**Severity**	**Mean PLR± SD**	**p-value**
Mild	150.78± 66.17	
Moderate	140.01± 55.24	
Severe	163.75± 72.46	0.419

**Table 5:** NLR trend according to severity (pre-treatment, 2 weeks, 1 month)

**Severity**	**Pre-treatment**	**2 Weeks**	**1 Month**
Mild	1.41± 0.45	1.27± 0.31	1.29± 0.20
Moderate	1.73± 0.79	1.52± 0.58	1.44± 0.44
Severe	4.60± 2.42	2.36± 1.01	1.59± 0.57

**Table 6:** Serum albumin trend according to severity (pre-treatment, 2 weeks, 1 month)

**Severity**	**Pre-treatment**	**2 Weeks**	**1 Month**
Mild	3.85± 0.43	3.99± 0.36	4.10± 0.33
Moderate	3.59± 0.43	3.74± 0.37	3.87± 0.31
Severe	2.99± 0.51	3.25± 0.43	3.49± 0.38
Repeated measures ANOVA
demonstrated statistically
significant improvement in
NLR and serum albumin across all
severity groups (p <0.01).

## References

[1] https://www.sciencedirect.com/science/chapter/edited-volume/pii/B9780323448871000262.

[2] https://www.ncbi.nlm.nih.gov/books/NBK482437/.

[3] Song X (2025). MedComm (2020)..

[4] Chen L (2017). Oncotarget..

[5] Im Y (2025). Tuberc Respir Dis (Seoul)..

[6] Martinez-Garcia MA (2017). Int J Chron Obstruct Pulmon Dis..

[7] Bhatt SP. (2024). Ann Am Thorac Soc..

[8] Donadio MVF (2020). J Thorac Dis..

[9] Ravi A (2025). Oral Sphere J. Dent. Health Sci..

[10] Liang J (2025). Ann Med..

[11] Gawaz M (2005). J Clin Invest..

[12] Georgakopoulou VE (2020). Cureus..

[13] Kwok WC (2024). Eur Clin Respir J..

[14] Soeters PB (2019). JPEN J Parenter Enteral Nutr..

[15] Paliogiannis P (2021). Clin Exp Med..

[16] Ju S (2021). Chron Respir Dis..

